# Enabling reusability of plant phenomic datasets with MIAPPE 1.1

**DOI:** 10.1111/nph.16544

**Published:** 2020-04-25

**Authors:** Evangelia A. Papoutsoglou, Daniel Faria, Daniel Arend, Elizabeth Arnaud, Ioannis N. Athanasiadis, Inês Chaves, Frederik Coppens, Guillaume Cornut, Bruno V. Costa, Hanna Ćwiek‐Kupczyńska, Bert Droesbeke, Richard Finkers, Kristina Gruden, Astrid Junker, Graham J. King, Paweł Krajewski, Matthias Lange, Marie‐Angélique Laporte, Célia Michotey, Markus Oppermann, Richard Ostler, Hendrik Poorter, Ricardo Ramírez‐Gonzalez, Živa Ramšak, Jochen C. Reif, Philippe Rocca‐Serra, Susanna‐Assunta Sansone, Uwe Scholz, François Tardieu, Cristobal Uauy, Björn Usadel, Richard G. F. Visser, Stephan Weise, Paul J. Kersey, Célia M. Miguel, Anne‐Françoise Adam‐Blondon, Cyril Pommier

**Affiliations:** ^1^ Plant Breeding Wageningen University & Research PO Box 386 Wageningen 6700AJ the Netherlands; ^2^ BioData.pt Instituto Gulbenkian de Ciência 2780‐156 Oeiras Portugal; ^3^ INESC‐ID 1000‐029 Lisboa Portugal; ^4^ Leibniz Institute of Plant Genetics and Crop Plant Research (IPK) Gatersleben 06466 Seeland Germany; ^5^ Bioversity International Parc Scientifique Agropolis II Montpellier Cedex 5 34397 France; ^6^ Geo‐Information Science and Remote Sensing Laboratory Wageningen University Droevendaalsesteeg 3 Wageningen 6708PB the Netherlands; ^7^ Instituto de Tecnologia Química e Biológica António Xavier Universidade Nova de Lisboa (ITQB NOVA) Avenida da República 2780‐157 Oeiras Portugal; ^8^ Instituto de Biologia Experimental e Tecnológica (iBET) 2780‐157 Oeiras Portugal; ^9^ Department of Plant Biotechnology and Bioinformatics Ghent University Technologiepark 71 Ghent 9052 Belgium; ^10^ VIB Center for Plant Systems Biology Technologiepark 71 Ghent 9052 Belgium; ^11^ Université Paris‐Saclay INRAE, URGI Versailles 78026 France; ^12^ BioISI – Biosystems & Integrative Sciences Institute Faculdade de Ciências Universidade de Lisboa Lisboa 1749-016 Portugal; ^13^ Institute of Plant Genetics Polish Academy of Sciences ul. Strzeszyńska 34 60‐479 Poznań Poland; ^14^ Department of Biotechnology and Systems Biology National Institute of Biology SI1000 Ljubljana Slovenia; ^15^ Southern Cross Plant Science Southern Cross University Lismore NSW 2577 Australia; ^16^ Computational and Analytical Sciences Rothamsted Research Harpenden AL5 2JQ UK; ^17^ Plant Sciences (IBG‐2) Forschungszentrum Jülich GmbH D‐52425 Jülich Germany; ^18^ Department of Biological Sciences Macquarie University North Ryde NSW 2109 Australia; ^19^ Department of Crop Genetics John Innes Centre Norwich Research Park, Colney Norwich NR4 7UH UK; ^20^ Oxford e‐Research Centre Department of Engineering Science University of Oxford 7 Keble Road Oxford OX1 3QG UK; ^21^ INRA, Laboratoire d'Ecophysiologie des Plantes sous Stress Environnementaux UMR759 Montpellier 34060 France; ^22^ Institute for Biology I BioSC RWTH Aachen University Worringer Weg 3 52074 Aachen Germany; ^23^ Royal Botanic Gardens, Kew Richmond TW9 3AE UK

**Keywords:** findability, interoperability, metadata, phenomics, plant phenotyping, reusability, standards

## Abstract

- Enabling data reuse and knowledge discovery is increasingly critical in modern science, and requires an effort towards standardising data publication practices. This is particularly challenging in the plant phenotyping domain, due to its complexity and heterogeneity.
- We have produced the MIAPPE 1.1 release, which enhances the existing MIAPPE standard in coverage, to support perennial plants, in structure, through an explicit data model, and in clarity, through definitions and examples.
- We evaluated MIAPPE 1.1 by using it to express several heterogeneous phenotyping experiments in a range of different formats, to demonstrate its applicability and the interoperability between the various implementations. Furthermore, the extended coverage is demonstrated by the fact that one of the datasets could not have been described under MIAPPE 1.0.
- MIAPPE 1.1 marks a major step towards enabling plant phenotyping data reusability, thanks to its extended coverage, and especially the formalisation of its data model, which facilitates its implementation in different formats. Community feedback has been critical to this development, and will be a key part of ensuring adoption of the standard.

## Introduction

The volume of data being generated in the life sciences demands good data management practices to enable reusability. While it is common practice to publish standardised sequencing data in public repositories, other data types are often only made available through scientific publications, and can be hard to find (Vines *et al.*, 2014), interpret or reuse. In a survey with over a 1000 participants, more than half agreed that lack of access to data is a ‘major impediment to progress in science’ and ‘has restricted their ability to answer scientific questions’, and most pointed out that data may easily be misinterpreted due to its complexity or poor quality (Tenopir *et al.*, 2011).

In the plant phenotyping domain, data reuse is both pressing and challenging (Spindel & McCouch, 2016; Tardieu *et al.*, 2017; Ćwiek‐Kupczyńska, 2018). On the one hand, the development of automated high‐throughput and high‐resolution technologies has contributed to a scale‐up in the number, complexity and size of plant phenotyping datasets. This has been amplified by the increasing number of long‐term, highly multilocal phenotyping networks aiming to decipher the interaction between genotype and environment (Millet *et al.*, 2019). Conversely, the reuse and meta‐analyses of phenotyping data are particularly challenging due to the heterogeneity of this domain that encompasses many types of experimental sites (field, glasshouse, controlled environment), plants (crops, forest trees), collected data (images, physical measurements, chemical assays, molecular biology assays), and experimental designs (factors being tested, timing, field layouts, etc.). Furthermore, plant phenotype hinges not only on the interaction between genotype and environment, but also developmental stage and epigenome status (King *et al.*, 2010), which raises the challenges of integrating genotypic and phenotypic data (Pommier *et al.*, 2019b).

A successful example of data reuse in this domain is the study by Hurtado‐Lopez *et al*. (2015), who reused field trial datasets and integrated them with quantitative trait locus (QTL) data to yield novel insights into genotype by environment (GxE) interactions in potato. Because the original experimental data followed no standardisation guidelines, the authors had to manually assemble detailed metadata during the preprocessing of the data from descriptions in unstructured text. To facilitate such studies, so that they may become the norm rather than the exception, it is essential that the scientific community adopt good data management and publication practices (Zamir, 2013).

The requirements for data reuse in science have been formalised in the FAIR data principles (Wilkinson *et al.*, 2016). They state the criteria that scientific data must fulfil to be findable, accessible, interoperable and reusable by both humans and machines, which hinge on having rich, harmonised, machine‐readable, high‐quality metadata describing the data as explicitly and objectively as possible.

Four key components are needed from research communities to meet these requirements: metadata standards which list the fields required for interpreting the data from a given experimental domain; machine‐readable (meta)data exchange formats in which to express and share the (meta)data; ontologies or controlled vocabularies to describe (meta)data values and ensure that they are objective, consistent and unambiguous across datasets; and searchable data repositories with a well established protocol for machine access.

The need for a metadata standard was first recognised in the life sciences by the microarray community, who developed MIAME (Minimum Information About a Microarray Experiment) (Brazma *et al.*, 2001). This was soon followed by similar standards for other domains (e.g. Field *et al.*, 2008; Bustin *et al.*, 2009; Lapatas *et al.*, 2015) as can be seen on FAIRsharing (Sansone *et al.*, 2019). In the plant phenotyping domain, the need for metadata to document experiments was initially addressed independently by the developers of phenotyping databases, such as BreedBase (BreedBase team, 2020), GnpIS (Steinbach *et al.*, 2013), PIPPA (PIPPA team, 2020) and Plant Hybrid Information System (PHIS) (Neveu *et al.*, 2019), which resulted in a multitude of implicit, often database‐specific standards. However, the need for an explicit consensus to enable interoperability between these databases brought this community together to develop MIAPPE (Minimum Information About a Plant Phenotyping Experiment), the first and so far only community metadata standard for the plant phenotyping domain (Krajewski *et al.*, 2015).

MIAPPE had three guiding principles: to minimise the chance of a researcher missing important information in the documentation of an experiment; to support the annotation of content with community‐relevant vocabularies; and to promote a data format implementation. MIAPPE marked a critical step towards the FAIRness of plant phenotyping data, as concluded in a survey of *c.* 50 citations of this standard in publications and web portals (Krajewski & Ćwiek‐Kupczyńska, 2020). However, there were aspects to improve, such as the coverage, usability and clarity of the standard. In particular, MIAPPE lacked fields needed to capture experiments with woody plants, as it was conceived primarily with crop plants in mind, and it lacked an explicit data model, which left some researchers struggling to understand how to represent their experiments.

The microarray community was again among the first to produce a machine‐readable (meta)data exchange format in the form of MAGE‐Tab (MicroArray Gene Expression tabular) (Rayner *et al.*, 2006), a standardised format for MIAME. This gave rise to the broader‐purpose ISA‐Tab (Investigation/Study/Assay tab‐delimited) format (Rocca‐Serra *et al.*, 2010; Sansone *et al.*, 2012), which was adopted by more domains, including plant phenotyping, with an ISA‐Tab implementation of MIAPPE (Ćwiek‐Kupczyńska *et al.*, 2016).

The use of ontologies and controlled vocabularies in the life sciences dates back to Linnaeus's taxonomy, but they have witnessed a more recent boom after the creation of the Gene Ontology (The Gene Ontology Consortium, 2019), and currently number in the several hundred, as seen on BioPortal (Noy *et al.*, 2009). For the plant phenotyping domain, there are a number of ontologies that cover different key aspects. The Crop Ontology (Shrestha *et al.*, 2012) models plant traits and methods for assessing them in several species‐specific ontologies. It merits special reference, in that it aims at standardising the methods used by data producers for phenotyping and the way they are reported, rather than only at terminological standardisation. It therefore includes an implicit metadata standard, the trait‐method‐scale trio, which was incorporated into MIAPPE. The Planteome project (Cooper *et al.*, 2018) developed three key ontologies: the Plant Trait Ontology (Arnaud *et al.*, 2012) modelling species‐independent plant traits under a broader scope than the Crop Ontology and serving as a reference ontology for multispecies analyses; the Plant Ontology (Jaiswal *et al.*, 2005) covering plant anatomical structures and development stages and enabling interplant comparisons; and the Plant Experimental Conditions Ontology (Cooper *et al.*, 2018) describing plant treatments. In addition to these, relevant ontologies include: the Agronomy Ontology (Aubert *et al.*, 2017) covering agronomic practices, techniques and variables; the Environment Ontology (Buttigieg *et al.*, 2013) describing natural environments; and the Statistics Ontology (Statistics Ontology Project, 2020) devoted to statistical methods. All these ontologies and several others are indexed in AgroPortal (Jonquet *et al.*, 2018) which serves as the reference repository and search service for plant‐related ontologies.

Finally, while searchable data repositories have long been the norm in the life sciences, especially concerning gene and protein data, only in the last decade has it become common practice to enable machine access to their data via application programming interfaces (APIs). Currently all major databases, such as GenBank (Benson *et al.*, 2013) or UniProt (The UniProt Consortium, 2019), provide such access, but most smaller databases do not. This was the case for the plant phenotyping domain up until recently, with its numerous, independent and heterogeneous local databases. To address this problem and enable interoperability between databases, the plant community undertook the development of the Breeding API (BrAPI) (Selby *et al.*, 2019), a common API for data search and retrieval that can be implemented by plant breeding databases irrespective of their internal data model. Like the databases it aims to connect, BrAPI also has an implicit (meta)data model that aims to reconcile the metadata available in existing databases, spanning organisational metadata, plant phenotypic (meta)data and genotypic (meta)data. BrAPI was initiated independently from MIAPPE, so while there is substantial overlap between the two resources, there are also a few key differences in their metadata fields, as well as differences in terminology.

The way forward for enabling FAIR plant phenotyping data lies in bringing together all of the components described above. MIAPPE would be the cornerstone of such an architecture, specifying the metadata that is needed and connecting metadata fields to the ontologies recommended to fill them, as well as reconciling the several implicit metadata models of existing knowledge resources. BrAPI would serve as the means for federating the many independent plant phenotyping databases to enable findability and accessibility, and should enforce and validate the MIAPPE compliance of datasets. The MIAPPE ISA‐Tab implementation would support data publication and exchange. And potentially all of the ontologies listed above would play a role in describing the data and metadata of plant phenotyping experiments in a standardised and unambiguous way. However, it is clear that further development effort on these resources is needed to attain such a goal.

In this paper, we detail the efforts of an international consortium to enhance the MIAPPE standard towards enabling FAIR plant phenotyping data. We describe the following refinements: (i) the extension of MIAPPE to accommodate a wider range of use cases (including those relevant to perennial and woody plants); (ii) the specification of a data model underlying the standard, to facilitate its interpretation and usage; (iii) the formalisation of MIAPPE in a computer‐interpretable format (using the Web Ontology Language, OWL) to enable dataset validation and computational analysis; and (iv) the alignment of MIAPPE and BrAPI to enable the exposure of MIAPPE‐compliant datasets via BrAPI endpoints.

## Materials and Methods

### Development of MIAPPE 1.1

To take on the challenge of improving MIAPPE, the community gathered both life and computer scientists. The former drove the documentation and description of the standard, ensuring that the terms and definitions are meaningful and not purely technical. The latter took the initiative for the technical aspects, involving data formalisation, organisation, integration, sharing and interoperability. This ongoing partnership ensures that MIAPPE bridges the domains of life and data science and addresses the needs of both communities.

The development of MIAPPE 1.1 was carried out collaboratively using simple and efficient protocols and format (spreadsheet). Throughout the process, drafts were presented and discussed with the international community through consultations by emails, the MIAPPE consortium GitHub issue tracker (MIAPPE Contributors, 2020a) and during ‘bring your own data’ training sessions.

Like its predecessor, MIAPPE 1.1 is a metadata standard that formally organises the documenting of a phenotyping dataset, including environmental aspects. It primarily structures the metadata, imposing no constraints on the data itself (which may consist of images, other binary data, tabular files, etc.).

In comparison with MIAPPE 1.0, MIAPPE 1.1 introduces several new concepts while preserving most of those already present. The major change, however, is that it moved from a simple checklist to a fully formalised data model that makes explicit mandatory information, restrictions and expectations and thus represents a major improvement in clarity from MIAPPE 1.0.

The key changes in MIAPPE 1.1 fall into one of three categories, which are detailed in the following subsections: scope extension, interoperability and data model specification. In addition to these, the MIAPPE data model has been formalised in OWL as the Plant Phenotyping Experiment Ontology (PPEO).

Note: throughout the rest of this document, we use *italics* to denote MIAPPE concepts, <angle brackets> to denote ontology concepts, and “double quotes” for MIAPPE field value examples.

### Scope extension

The scope of MIAPPE 1.0, mostly restricted to field crops, was extended in MIAPPE 1.1 to encompass woody plants, mainly by enabling the identification of plant materials by their geolocation coordinates, which are typically used to identify forest trees instead of plant identifiers (e.g. GenBank accession numbers) used in crop research. Two levels for plant material identification and description are available in MIAPPE 1.1: (i) its identification within the experiment (*biological material*); and (ii) its identification before the experiment (*material source*), allowing for individual plants, lots or progeny to be described and related to previously published or publicly accessible material. Furthermore, the *preprocessing* field (previously called *pretreatments*) can describe any type of action performed on the *material source* before it is used as the experimental *biological material* (for instance “tree transplantation” and “grafting”).

### Interoperability

MIAPPE 1.1 incorporates several metadata standards and practices that cover parts of its scope, in order to ensure interoperability and avoid remodelling and redefining aspects that are already well established: the generic metadata fields (e.g. identifier, title, version, date) in MIAPPE 1.1 are largely based on the DataCite metadata model of Dublin Core (DataCite Metadata Working Group, 2014); the fields for biological material identification are based on the Multi‐Crop Passport Descriptors (mcpd) v.2.1 (Alercia *et al.*, 2015); the observation unit concept and its fields were imported from BrAPI and GnpIS‐Ephesis (Pommier *et al.*, 2019b); and the observed variable section is largely based on the data model of the Crop Ontology.

Additionally, to further foster interoperability, MIAPPE 1.1 includes precise definitions and examples for each of its fields, with recommendations for the use of controlled vocabularies, ontologies and ISO norms whenever appropriate. For example, the ISO 8601 norm is recommended for dates, Crop Ontology terms are recommended in the observed variable section, and Plant Ontology terms are recommended for characterising samples. These definitions and recommendations clarify the intended usage of MIAPPE 1.1 in a way that is accessible to biologists and breeders, while promoting compliance with the FAIR principles.

### Data model specification

The specification of a data model was essential to clarify MIAPPE's structure, and improves its internal consistency. The construction of a formal model helped: (i) ascertain the roles and relationships of the MIAPPE 1.0 checklist's main categories and concepts; and (ii) extend those concepts to a broader range of experiments.

**Fig. 1:**
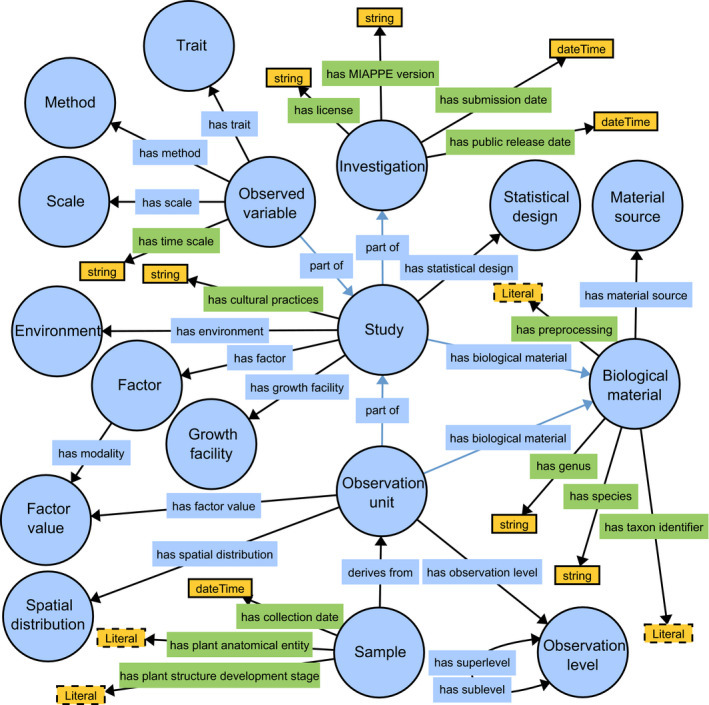
Subset of the Plant Phenotyping Experiment Ontology representing the MIAPPE data model. Generated using WebVOWL (http://editor.visualdataweb.org/) and edited manually. Circles indicate classes. Object properties are shown in blue rectangles, and data properties are shown in green rectangles. Yellow rectangles represent literals.

Objects in the MIAPPE 1.1 data model correspond to sections in the MIAPPE 1.1 checklist. A schematic view of the data model is presented in Fig. 1.

The MIAPPE data model is reconciled with the more generic data models underlying the ISA‐Tab exchange format (Rocca‐Serra *et al.*, 2010) through key objects such as Investigation and Study (ISA) and specialised representations such as BrAPI (Selby *et al.*, 2019) with entities such as Observation unit and Observation variable (BrAPI).

### Data model formalisation (PPEO)

While the specification of the MIAPPE data model addresses the concern of improving the clarity of the standard for users, it does not address machine readability, which is important to enable validation and facilitate implementation at scale. The need for the latter led us to encode the MIAPPE standard in OWL as the PPEO (Pommier *et al.*, 2020).

In PPEO, each MIAPPE section is encoded as an ontology class, with additional classes declared to group linked MIAPPE fields (e.g. <method> groups the linked fields *method description* and *method accession number*). Each MIAPPE field is encoded as an ontology data property, which specifies the type of value expected (e.g. <has collection date> for class <sample>, which must take a date–time value). The relations between classes are formalised through object properties (e.g. <has biological material> connects <observation unit> to <biological material>). Cardinality restrictions imposed by the data model are encoded as ontology restrictions on the corresponding classes (e.g. an <investigation> must have at least one <study>). Ontology usage recommendations are encoded as annotations. Finally, to facilitate the implementation of MIAPPE in various forms, PPEO includes labels expressing corresponding names of each class in different resources (BrAPI, ISA‐Tab).

Fig. 1 represents a subset of the PPEO.

### MIAPPE 1.1 overview

The MIAPPE sections, which correspond to objects in the data model, are the following:

- *Investigation* – the entry point of each MIAPPE dataset. It contains several general metadata fields (e.g. *title*, *description*, *submission*/*publication dates*), including some critical for FAIRness (*unique identifier*, *licence*, *MIAPPE version*). One or more *publications* may be associated with the *investigation*.
- *Study* – corresponds to one experiment and defines its location (which by definition must be single per *study*) and duration. It lists general fields documenting the experiment (e.g. *experimental design*, *cultural practices*, *growth facility*). Like the *investigation*, it contains a *unique identifier* field. An *investigation* must have one or more *studies*.
- *Person* – contains contact details for each contributor of an entire *investigation* or an individual *study*, including the *role* of the *person*.
- *Data file* – references a data file of the MIAPPE dataset (e.g. a tabular file containing the results of observations, an image file), which may be attached to the dataset (referenced by name) or available in an online repository (referenced by URL). A *version* and a *description* must be provided for each *data file*. A *study* may have any number of *data files*.
- *Biological material* – identifies and describes the plant materials used in the *studies*. Plant materials must be identified through a *biological material ID* field, which can be institution‐specific or platform‐specific (e.g. seed lot number for annual plants, clone number for perennials or an experimental plant ID), and is recommended to follow the MCPD convention of holding institute identifier (FAO WIEWS code) plus a unique identifier of the individual plant material provided by that institute. They must also be identified through the *organism* field, which indicates the unique taxonomic identifier of the *biological material* in a standard such as the NCBI taxonomy. Optionally, they may be identified through the fields *genus*, *species* and *infraspecific name*, where textual names are expected (but should follow accepted standards). They may also be identified through geographical coordinates (i.e. *latitude*, *longitude*, *altitude*, and *coordinates uncertainty*), as is common for forest trees. The *biological material preprocessing* describes the *biological material* pretreatments, applied (e.g. to the seeds, or the tree cuttings) before the beginning of the experiment. Finally, the *material source* fields identify the origin or provenance of the *biological material* (e.g. gene bank accession, *in situ* material like an orchard, tree material provenance including forest wild site, laboratory‐specific populations). These fields include the *material source ID* (which follows the same recommendations as the *biological material ID*), the *material source DOI* (for referencing material sources listed in repositories), four geographical coordinates fields (same as for *biological material*), and finally a textual description. The *biological material* section thus covers a minimal subset of the MCPD standard used by gene banks, while also enabling interoperability and data linking through the use of identifiers (namely NCBI taxonomy identifiers) both between MIAPPE‐compliant datasets and with external datasets. Moreover, through the provision of external identifiers to resources detailing their *biological material* (e.g. DOIs, accessions to gene banks or genome archives) researchers can encompass additional information, such as extended MCPD information (e.g. synonyms, genealogy) and genotypic information. Last but not least, with these additions, MIAPPE 1.1 can handle cases such as forest tree clonal trials, where the plants identified solely through *biological material* coordinates in one *study* are used to generate new plant material for another *study*, in which their identification is done by specifying the location of the *material source*.
- *Environment* – describes a management practice parameter (e.g. sowing density, rooting medium composition) that was kept constant throughout the *study* across all *observation units* (to be described later). It applies to the whole *study* and has only a type (*parameter*) and a *value*. There can be discrepancies between intended environmental settings (e.g. target temperature in a glasshouse) and actual measurements of environmental *observed variables* (e.g. hourly temperature measured with four sensors). A *study* may have any number of *environments*.
- *Experimental factor* – describes a management practice that varied between *observation units* in a *study*, assessing the effect of which is the object of the *study*. *Experimental factors* can be biotic or abiotic (e.g. pest, disease interaction, cultural practice) and are characterised by a *type*, a *description* and a list of possible *values*. For instance, a “drought” *experimental factor* can discriminate “rainfed” and “irrigated” blocks, and a “nitrogen input level” can identify groups of plants under “high nitrogen input”, “low nitrogen input” and “no nitrogen inputs”. A *study* may have one or more *experimental factors*.
- *Event* – describes a discrete occurrence at a specific time that affected the whole *study* or one or more *observation units*, which can be the application of a field/glasshouse practice (e.g. planting, fungicide application) or an unpredictable happening (e.g. rainfall, pathogen attack). *Events* allow a general traceability of the conditions/events, and have been adopted since their usefulness was successfully demonstrated in the PHIS (Neveu *et al.*, 2019). *Events* include a *type*, ideally taken from an ontology such as the Crop Research Ontology (Shrestha, 2020) or the Agronomy Ontology (Aubert *et al.*, 2017), a *date* and a *description*, but no dedicated field for categorical or numerical values. *Events* can be repeated through time (e.g. to capture repeating cultural practices, such as adding fertiliser) by duplicating the *type* and *description* while providing a new *date*.
- *Observation unit* – is the experimentation object on which phenotypic and environmental parameters are measured and to which *experimental factors* are applied. It is characterised by a *type* or level, which can be a single “plant”, a group of plants (“pot”, “plot”, “block”), or the whole “study”. These *types* are hierarchical, meaning that we can have *observation units* and corresponding observations made from the *study* level down to the plant level. In some cases, an *observation unit* may contain no plant (e.g. raw plots after harvest or areas of a forest without tree), but can still be the object of environmental observations. Optionally, an *observation unit* can have a cross‐reference to an external database, such as BioSamples (Courtot *et al.*, 2019). Also optionally, it can have one or more *spatial distribution* key‐value pairs that locate the *observation unit* in the experimental hierarchy (e.g. “block: 1”) or globally (e.g. “latitude: +43.619261”). A *study* should have one or more *observation units*.
- *Sample* – represents subplant material that was physically collected from an *observation unit* and was stored and processed before observations are made on it (e.g. in molecular studies). When traceability of *sample* processing is not needed, subplant observations can be assigned directly to the corresponding plant‐level *observation unit* without the use of a *sample* as an intermediary. In such cases, the *observed variable* should describe that the observation is made on a plant part (e.g. “leaf chlorophyll content”, “grain protein content”) and include additional information on how the sampling was made in the textual description. The *sample description* field contains a free text description such as organism count, oxygenation, salinity or storage attributes. The *plant anatomical entity* and the *plant structure development stage* give more details on the *sample* properties at the time of sampling, which is specified with the field *collection date*. A *sample* must be derived from a single *observation unit*, but each *observation unit* may have any number of derived *samples*.
- *Observed variable* – documents a phenotypic or environment parameter that was observed and recorded as part of the *study*. It follows the Crop Ontology model of representing variables as combinations of a *trait*, a *method* and a *scale*. *Trait* details the characteristic being observed/measured (e.g. “plant height”). *Method* describes the procedure used in the observation/measurement (e.g. “with a measuring tape, starting at ground level”). *Scale* indicates the unit or scale with which observations/measurements were recorded (e.g. “cm”). *Observed variables*, *traits*, *methods* and *scales* are each identified by name, and may have a reference to the corresponding ontology concept (ideally from the Crop Ontology). *Observed variables* also have an *ID* by which they are referenced in the data file. *Methods* can also have a *description* plus an *additional reference*, usually from the literature. The *time scale* indicates the unit of time (e.g. “date–time”, or “growing degree days”) used to timestamp observations of this *observed variable*.

Note that in MIAPPE 1.1, the description of environment aspects is broken into several sections so as to allow flexibility in capturing and representing environment information: *environment* (fixed parameters throughout the *study*), *experimental factors* (fixed set of values for the *study* which vary between *observation units*), *observed variables* (measured during the *study*) and potentially *events* (discrete occurrences such as heavy rain).

### MIAPPE implementations

MIAPPE is a general specification that needs to be adopted and implemented by data repositories and exchange tools if it is to be easily usable. The MIAPPE 1.1 update encompasses four major implementations which are discussed in the following subsections: (i) an ISA file archive backed by an updated ISA‐Tab configuration, developed in collaboration with the ISA Framework team; (ii) a web service implementation through BrAPI, developed in close collaboration between the MIAPPE and BrAPI communities; (iii) a spreadsheet template developed and used as training material to introduce biologists to MIAPPE, which can also be used for simple metadata exchange; and (iv) finally, an RDF implementation based on the PPEO.

### MIAPPE ISA‐Tab

The ISA Framework encompasses a model and a set of serialisations (TAB, JSON and RDF) to describe the experimental metadata with links to data files, code, articles and other digital objects. It comes with a suite of associated tools, is extensively used in the life sciences (The ISA Team, 2020), and among the endorsed resources of the ELIXIR Interoperability Platform.

MIAPPE 1.0 already included an ISA‐Tab implementation in the form of a configuration file. This implementation has been revised in view of the changes in MIAPPE 1.1, and the configuration file has been updated accordingly. This configuration (ISA‐Tab for Plant Phenotyping Contributors, 2020) can be used with the ISA Creator tool, to more easily produce MIAPPE‐compliant ISA‐Tab archives.

The overview of the mapping between MIAPPE 1.1 and ISA‐Tab sections is shown in Table 1. The Investigation and the Study express the same concepts in both MIAPPE and ISA‐Tab, and many of their fields are listed under the corresponding sections. There are also direct correspondences for *experimental factors* (Study Factors), *biological material* (Source), *observation units* (Samples) and *samples* (Extracts). The remaining MIAPPE‐specific fields are stored as ISA‐Tab Comments. ISA Protocols must include a protocol named “Growth” holding the MIAPPE *cultural practices* field and *environment parameters*, one protocol for the “Phenotyping” process, plus an optional list of protocols with Type “Event” to handle MIAPPE *events*, with specific occurrences listed in an external Events file. Finally, the ISA Sampling Protocol indicates the derivation of a MIAPPE *sample* from an *observation unit*. Each ISA‐Tab Assay represents one data file measured at one observation level. *Observed variables* are listed in the trait definition file, and referenced in the data files. The data files are formatted according to the common practices of the domain and contain references to that *Variable ID*, the measured values and times plus any information which researchers might deem useful.

**Table 1:** Mapping between MIAPPE and ISA‐Tab sections.

MIAPPE section	ISA‐Tab section	ISA‐Tab section specification
Investigation	Investigation/investigation publications	
Study	Study/study design descriptors/study protocols	
Person	Investigation contacts/study contacts	
Data file	Study	With comment fields
Biological material	Source	
Environment	Study protocols	Growth type protocol
Experimental factor	Study Factors	
Event	Study protocols	Event type protocols and external Events file
Observation unit	Sample	
Sample	Extract/study protocols	Sampling type protocol
Observed variable	Observed variable	In external trait definition file

The table lists the MIAPPE sections with the ISA‐Tab sections holding their fields. MIAPPE‐exclusive fields have been added as comments in the corresponding sections. The detailed mapping can be found in Supporting Information Table S1, and in the MIAPPE repository (https://github.com/MIAPPE/MIAPPE/tree/master/MIAPPE_Checklist‐Data‐Model‐v1.1/MIAPPE_mapping).

### Breeding API (BrAPI)

The Breeding API (BrAPI) (Selby *et al.*, 2019) is a RESTful API developed by an international open‐source community for querying plant breeding data, already implemented by several databases, and selected by the European biological data infrastructure ELIXIR as the cornerstone of its plant data search service. It is therefore critical that BrAPI and MIAPPE be compatible.

The collaboration between the BrAPI and MIAPPE teams has aimed at ensuring the compatibility of the two schemas, and the latest brapi (v.1.3) covers most of MIAPPE. The main sections correspond to the same concepts and either share the same name or have direct correspondence, such as *investigation* (BrAPI Trial) and *biological material* (BrAPI Germplasm). Some MIAPPE sections are currently absent from BrAPI (e.g. *environment*, *event*) but have already been proposed as additions to BrAPI and are under consideration for the next major release. The mapping between MIAPPE and BrAPI is overviewed in Table 2.

**Table 2:** Mapping between MIAPPE sections and BrAPI objects.

MIAPPE	BrAPI object
Investigation	Trial
Study	Study
Person	Contact
Data file	Data link
Biological material	Germplasm
Environment	Environment parameter
Experimental factor	Treatment
Event	Events
Observation unit	Observation unit
Sample	Samples
Observed variable	Variable

The table lists the MIAPPE sections with the BrAPI objects holding their fields (in the current and future versions). The detailed mapping for each field can be found on the MIAPPE GitHub repository and in Supporting Information Table S1, and in the MIAPPE repository (https://github.com/MIAPPE/MIAPPE/tree/master/MIAPPE_Checklist‐Data‐Model‐v1.1/MIAPPE_mapping).

Finally, BrAPI datasets can be exported as MIAPPE‐compliant ISA‐Tab archives using the BrAPI2ISA tool (BrAPI2ISA contributors, 2020).

### Spreadsheet template

The spreadsheet template for MIAPPE (MIAPPE Contributors, 2020b) was developed mainly for training purposes, as a simpler alternative to ISA‐Tab. It is an explicit representation of MIAPPE, where each section has been placed in a separate worksheet. This template facilitates the understanding of the connections between documentation, data model and actual data. For training, it is important that the data model and one‐to‐many relationships (Fig. 1) be explicitly presented and comprehensively explained to the users (e.g. biologists or data managers).

### RDF based on the PPEO

PPEO was conceived to enable the direct expression of MIAPPE datasets in RDF (W3C, 2020a), by instantiating the ontology. Moreover, because PPEO explicitly maps MIAPPE to its implementations, it should be straightforward to convert MIAPPE datasets expressed in any of them to RDF.

MIAPPE and BrAPI are also connected through PPEO, which not only maps the two resources, but also includes classes exclusive to BrAPI, such as the <observation> class (which is outside of the scope of MIAPPE, as it pertains to data). A proof of concept has demonstrated the feasibility of producing linked data through BrAPI using the JSON‐LD format (W3C, 2020b), with PPEO enabling the semantic mapping (see this dataset: Oury *et al*. (2020)).

Having MIAPPE datasets in RDF enables the use of a wide range of available tools for reasoning and analysis, and facilitates data integration (by enabling data linking and cross‐referencing at the semantic level) and validation.

## Results

To evaluate the applicability of the standard and the functionality of its implementations, plant scientists were asked to describe their phenotyping experiments using MIAPPE 1.1. The datasets were provided by: the Instituto de Biologia Experimental e Tecnológica (iBET), Portugal; the Leibniz Institute of Plant Genetics and Crop Plant Research (IPK), Germany; the Genetic and Genomic Information System (GnpIS) of the Institut National de la Recherche Agronomique, France; and the Vlaams Instituut voor Biotechnologie (VIB), Belgium. The datasets are summarised in Table 3, and described in detail in Supporting Information Notes S1. All of them are listed under Papoutsoglou *et al*. (2019), and their files can be retrieved through the repositories listed there (Baute *et al*., 2019a, 2019b, 2019c, 2020; Chaves *et al*, 2019a, 2019b; Junker, 2020; Junker & Li, 2020; Michotey, 2019, 2020; Oury *et al*., 2019a, 2019b; Pea *et al*., 2019a, 2019b).

**Table 3:** Overview of some characteristics of the example datasets.

Publication	Inácio *et al*. (2017)	Junker *et al*. (2015)	Li *et al*. (2019)	Oury *et al*. (2018)	Monclus *et al*. (2012)	Baute *et al*. (2015)	Dell'Acqua *et al*. (2015)	Baute *et al*. (2016)
Biological material	Natural population trees identified by geographical location; material source not identified Population	Mutant; multiple replicates RIL Population	Mutant and wild‐types	Genebank material	Clonal material with *material source* traceability; includes crosses and populations	RIL	Population	RIL
Throughput	Low	High	High	Low	Low	High	Low	High
Plant type	Forest tree	Model plant	Crop	Crop	Forest tree	Crop	Crop	Crop
Setting	Field; three locations	Automated glasshouse, controlled environment, four experimental factors	Automated glasshouse	Multiyear multilocal field network	Field	Glasshouse	Field	Glasshouse
Field Dataset	Glasshouse Cork oak	*Arabidopsis*	Barley	Wheat	Poplar	Maize	Maize	Maize

The experiments on the table encompass different experimental settings, plant types and throughput. RIL, recombinant inbred line.

The datasets span model, crop and perennial plants in a variety of experimental settings, as well as various MIAPPE 1.1 implementations. They demonstrate the ability of MIAPPE to handle diverse experimental designs, including automated glasshouses (IPK and VIB datasets), field networks for crops (GnpIS) and forest trees (iBET and GnpIS) with multiple scales and repetitions. Perennial plant use cases feature time series data, that is several observations across time for the same *observed variable* on the same plant. Field networks (GnpIS wheat) demonstrate the use case of a multilocal and multiannual dataset where each location represents one *study* over several years. Several datasets demonstrate also the use of *experimental factors* such as cultural practices (nitrogen level in GnpIS wheat) or experimental questions (covered or uncovered plants in IPK *Arabidopsis*). The *observed variables* proved to be well suited for very diverse destructive and nondestructive measurements adapted to agronomic (e.g. yield, grain weight), morphological (e.g. plant height), stress (e.g. disease or game), molecular (e.g. protein content) and physiological (e.g. photosynthetic efficiency) data. The data types covered by the *observed variables* are mostly numeric or textual, but also include images (IPK barley). These *observed variables* were described using references to ontologies (Michotey *et al.*, 2019; Pommier *et al.*, 2019a; Michotey & Chaves, 2020) whenever possible, but ad hoc variables were also used in specific cases not covered by ontologies. One of the most challenging aspects addressed by MIAPPE and successfully demonstrated by the datasets is the documentation of the *biological material*. The datasets clearly demonstrate how to organise information for model plants (IPK *Arabidopsis*), mutants (IPK barley), recombinant inbred line and population (VIB maize), GenBank reference accessions (GnpIS wheat) and perennial plants including *in situ* material (iBET cork oak stands) or dedicated experimental locations acting as experimental tree fields with populations or crosses (GnpIS poplar).

While the datasets showcase the applicability of MIAPPE to diverse experimental settings, they by no means represent the full extent of its coverage. Additional settings that were contemplated in the conception of MIAPPE but are not covered by the examples include: high‐throughput phenotyping facilities with plants manipulated by conveyor belts, which produce large volumes of data with respect to the positions of plants and their development; precision agriculture field studies with drones and sensors capturing a wealth of data both about plant development and the environment; and cases where tracing the identity of plant materials is more complex. In the interest of demonstrating MIAPPE's coverage, Table 4 presents additional examples of settings and details their modelling in MIAPPE.

**Table 4:** Modelling possibilities for complicated experiment details.

No.	Scenario	MIAPPE modelling
(1)	Heterozygous parent genotypes are used to derive a crossing population exhibiting significant phenotypic segregation. Genotype tracing is necessary.	The cross of the parents is mentioned in the *material source*. Each of the progeny is treated as a *biological material* derived from the same *material source*, and is attributed a unique ID.
(2)	Each tree in a field is observed through several sensors, at the roots and near its top.	*Observation unit levels*: “plant”. Each tree is a single *observation unit*. Each sensor measures one or many o*bserved variables*, (e.g. “Canopy temperature”, “Cork thickness”, …)
(3)	A sensor is placed in the middle of the field.	An *observation unit* is created for the sensor. No plants have to be present for an *observation unit* to be valid, as long as that *observation unit* is used to produce measurements or express *experimental factor values*.
(4)	Multilocal, multiyear field phenotyping network	*Observation unit levels*: “study”> “genotype”> “plot”. The whole network is an *investigation.* Each location is a *study* over several years. The *biological material* list is shared for the whole *investigation*. The list of *observed variable* definitions is also shared by all *studie*s. The measured data and observations can be at the “plant” or “plot” level, or as a per‐genotype average within each study. Study‐level observations can be measurements from a meteorological station.
(5)	Time series of event or observation.	*Observation unit levels*: any. *Study* type: any. *Observed variables* list the *time scale* they use. In the data file, a single *observed variable* is measured several times, each value being timestamped in julian days, growing degree days or any other time scale. The same applies with *events* with a given *event type* recorded several times at different time stamps.

The table shows more specific scenarios that may be necessary to accommodate in MIAPPE, and the proposed modelling for them inside the standard.

## Discussion

A global metadata standard is a key component for enabling FAIR data in any research domain, by providing a common framework under which researchers can describe their datasets with the necessary information for their interpretation, thus promoting interoperability and reusability. MIAPPE aims at serving such a role for the plant phenotyping community, and the first version of the standard took ample strides in that direction. In this work, we summarise the steps taken to extend and improve the usability of the standard.

The datasets presented in the Results demonstrate the broader applicability of MIAPPE 1.1, which was one of the main goals behind the update. The datasets span a variety of settings (e.g. woody, crop and model plants; glasshouses, single fields and field networks; single‐year and multiannual experiments) and include aspects that could not be modelled under MIAPPE 1.0 (the most critical being the identification of *biological materials* using geographical coordinates).

MIAPPE 1.1 also has improved in flexibility and usability compared to the previous version. It has clearer definitions, examples, and when applicable, ontology recommendations for all fields. It has an explicit data model available in schematic form and encoded in OWL as PPEO. It has fewer mandatory fields, since not all of them are applicable to all experiments. It allows different strategies for modelling aspects such as environmental parameters: under the *environment* section, as *events* or as *observed variables*. The improved *biological material* description and the new *material source* can now handle gene banks and experimental collections with either the bare minimum identification or very detailed information, including infraspecific description, provenance, complex processing or identification mechanism. We received evidence for the improved usability of MIAPPE from the community, during two open requests for feedback and in several training sessions. The specification of the data model and the enriched definitions and examples were highlighted as clear improvements.

While MIAPPE promotes interoperability and reusability, the other two FAIR principles (findability and accessibility) rely mainly on BrAPI, which enables data search and retrieval through machine access. However, for these two resources to be part of a common scheme for enabling FAIR plant phenotyping data, it is necessary to ensure that BrAPI calls adequately cover MIAPPE and enable searches by all key MIAPPE fields. The process of reconciling MIAPPE and BrAPI was undertaken in parallel with the MIAPPE 1.1 update, through a collaboration between the BrAPI team, the ELIXIR Interoperability platform and Plant Sciences community, the EMPHASIS Plant Phenotyping Infrastructure and the CGIAR. BrAPI will be fully MIAPPE 1.1 compliant once its (currently beta) 2.0 release is finalised.

This reconciliation and the interoperability between the various MIAPPE 1.1 implementations is demonstrated in our Results, as most datasets are available in two different implementations (including BrAPI), in many cases through automatic conversion. This is critical, as MIAPPE aims to support a wide range of users and applications, from data submission by life scientists to data exchange, validation and even reasoning by machines. Formats supporting all these applications must not only be available but also be interconvertible.

While, from a technical standpoint, we believe that the merits of MIAPPE 1.1 speak for themselves, we are well aware of the many hurdles ahead of getting any standard widely adopted by the community it seeks to serve. Indeed, there are several dozen standards currently deprecated in the FAIRsharing portal and surely many more have been lost to history.

One of the main factors behind wide adoption is having community engagement throughout the development process. Indeed, GO (The Gene Ontology Consortium, 2019) has been so successful because it emerged from the communities involved in gene function annotation for several model organisms and has remained open to input from the community throughout its history. The story behind MIAPPE is curiously similar, as its development gathered several researchers involved in plant phenotyping repositories, the process of updating it had extensive direct engagement with the community, and it remains open to community input through its GitHub repository (MIAPPE Contributors, 2020a). MIAPPE 1.1 therefore gathers as close to a community‐wide consensus as is possible to get and distil into a clear and well‐organised standard, especially considering the heterogeneity and complexity of the plant phenotyping domain, and the difficulty in reconciling the perspectives of its different subdomains and experiment types. We will further foster its adoption through constant efforts of outreach and dissemination, to gather new communities and ensure the long‐term usefulness of the standard.

Also critical for adoption is demand: when funders and/or publishers require compliance with a standard or data publication practice – such as depositing sequencing data in one of the public gene banks – it tends to be widely adopted. In the case of MIAPPE, the demand consists of the increasing pressure from funding agencies towards compliance with the FAIR data principles. Researchers working in plant phenotyping and seeking FAIR data solutions will be pointed towards MIAPPE thanks to its presence in the FAIRsharing portal (FAIRsharing.org: MIAPPE, 2020) and above all to the endorsement of ELIXIR, which led the MIAPPE 1.1 update and is helping shape the policies and lay the foundations needed for enacting the FAIR principles.

Equally critical is usability, as researchers tend to view the need for standardisation and reusability as a burden and often do as little effort as they can get away with when submitting a dataset, unless the process is virtually effortless. While MIAPPE's usability was improved with the 1.1 update, it is still missing an easy‐to‐use submission interface. For this reason, we are engaging with popular data management tools such as COPO (The COPO team, 2020) or FAIRDOM (Wolstencroft *et al.*, 2017) to incorporate MIAPPE and thus enable user‐friendly MIAPPE‐compliant dataset submission.

Last but not least, in order to persist, a standard must constantly evolve to keep up with technical and scientific advances. In this regard, the 1.1 update demonstrates that MIAPPE is very much a living standard, and we are already starting the next phase of development of MIAPPE. It will concentrate on two main aspects: extending its coverage of environmental aspects (initiated by the EMPHASIS members of the MIAPPE community) and facilitating the recording of technical aspects of material and data processing (e.g. sensors, cameras, software, configurations, calibrations), which are becoming increasingly important. Within this scope, another possible improvement could be to establish a formal complementarity with the ICASA standard, which is used by the agronomic and modelling communities, and provides variables for agronomic management practices, treatments, environmental conditions and measurements of crop responses (White *et al.*, 2013). There is overlap between these two standards – with MIAPPE dedicated to plant phenotyping as used by geneticists, biologists and some agronomists and ICASA to ‘any field experiment or crop production situation’ – but the scope of each extends far beyond that of the other and there are obvious complementarities between them. User feedback from these endeavours will steer further developments, by revealing areas where improvement is desired by the community.

## Author contributions

EAP, DF, DA, EA, INA, IC, FC, GC, BVC, HC‐K, BD, RF, KG, AJ, GJK, PK, ML, M‐AL, CM, MO, RO, HP, RR‐G, ŽR, JCR, PR‐S, S‐AS, US, FT, CU, BU, RGFV, SW, PJK, CMM, A‐FA‐B and CP contributed to the development of MIAPPE 1.1, participating in the discussions and responding to the public request for comments. A‐FA‐B, BD, CMM, CP, DA, DF, EAP, FC, GC, HC‐K, IC and PR‐S contributed to the MIAPPE implementations. A‐FA‐B, AJ, BVC, BD, CM, CMM, CP, DA, FC, IC, ML and US contributed to the evaluation of the new version of MIAPPE. EAP coordinated the drafting of the manuscript, with contributions from A‐FAB, CMM, CP, DF and IC. A‐FA‐B, CMM, CP and PJK coordinated the research. All authors read, provided feedback and approved the final manuscript.

## Supporting information


**Notes S1** Summaries of the datasets used to evaluate MIAPPE 1.1.

**Table S1** Detailed mapping between MIAPPE, ISA‐Tab and BrAPI fields.
Please note: Wiley Blackwell are not responsible for the content or functionality of any Supporting Information supplied by the authors. Any queries (other than missing material) should be directed to the *New Phytologist *Central Office.
