# Supplementary material for: Enabling reusability of plant phenomic datasets with MIAPPE 1.1
Source: New Phytol. 2020 Apr 25;227(1):260–73. doi: 10.1111/nph.16544 (PMC7317793; doi:10.1111/nph.16544)
Supplement: Supplementary file 1 — Notes S1 Summaries of the datasets used to evaluate MIAPPE 1.1. [file NPH-227-260-s001.pdf]

New Phytologist

New Phytologist Supporting Information

Article title: Enabling reusability of plant phenomic datasets with MIAPPE 1.1

Authors:
Evangelia A. Papoutsoglou, Daniel Faria, Daniel Arend, Elizabeth Arnaud, Ioannis N. Athanasiadis, Inês Chaves, Frederik Coppens, Guillaume Cornut, Bruno V. Costa, Hanna Ćwiek-Kupczyńska, Bert Droesbeke, Richard Finkers, Kristina Gruden, Astrid Junker, Graham J. King, Paweł Krajewski, Matthias Lange, Marie-Angélique Laporte, Célia Michotey, Markus Oppermann, Richard Ostler, Hendrik Poorter, Ricardo Ramírez-Gonzalez, Živa Ramšak Jochen C. Reif, Philippe Rocca-Serra, Susanna-Assunta Sansone, Uwe Scholz, François Tardieu, Cristobal Uauy, Björn Usadel, Richard G.F. Visser, Stephan Weise, Paul J. Kersey, Célia M. Miguel, Anne-Françoise Adam-Blondon, Cyril Pommier

Article acceptance date: 24 February 2020.

The following Supporting Information is available for this article:

Table S1 Detailed mapping between MIAPPE, ISA-Tab and BrAPI fields. **(see separate file:**

MIAPPE_v1.1_full_mapping.pdf)

Notes S1 Summaries of the datasets used to evaluate MIAPPE 1.1.

Notes S1:

All datasets and files mentioned in this supporting information file are listed and accessible via the accompanying supplementary dataset (Papoutsoglou et al., 2019).

Notes S1.1 Cork oak dataset (iBET)

The cork oak dataset derives from a report by Inácio et al. (2017) and focuses on the evaluation of cork quality traits of cork oak (*Quercus suber*) trees and the putative correlation between those traits and DNA methylation in living cork cells. This *investigation* includes three *studies* corresponding to three cork oak stands in different locations in Portugal, characterized by Costa et al. (2016). In each *study*/stand, 8 to 10 trees were randomly chosen, and in total 27 trees were assessed. For each tree, 20 cork quality traits were evaluated after debarking by manually phenotyping the cork plank. All the *traits* were described using the Woody Plant Ontology (Michotey et al., 2019; Michotey & Chaves, 2020).

As is typically the case, the cork oak trees in this dataset are identified only by means of their geographical coordinates. In this dataset, the *material source* identification is not described

because cork harvesting for industrial applications starts on trees that are over 40 years old, and the life history of trees that old is often lost or unknown.

The dataset is available from the PHENO BrAPI endpoint (Chaves et al., 2019b), and in the spreadsheet template in Chaves et al. (2019a).

Notes S1.2 Arabidopsis dataset (IPK)

The *Arabidopsis* dataset is the result of an investigation of movement and soil cover effects on plant growth in a high throughput plant phenotyping system which combines a growth chamber for controlled environmental conditions and the imaging chambers for non-invasive trait assessment (Junker et al., 2015). *Arabidopsis thaliana* plants were grown with a large number of replicates and their growth and development was evaluated with respect to two factors: *i)* "moving vs. stationary" to assess if the movement of plants on the conveyor belt influences plant growth and *ii*) "covered vs. uncovered" to assess if soil covers influence plant growth. These special soil covers are used for reducing transpiration and to facilitate segmentation of plant pixels from the background during image analysis. The dataset is an update of a previously published version (Junker et al., 2020) based on MIAPPE 1.0 (Arend et al., 2016b).

The dataset was encoded in ISA-Tab, uploaded to the Plant Genomics and Phenomics repository (Arend et al., 2016a) and is available at Junker (2020).

Notes S1.3 Barley dataset (IPK)

The barley dataset is the result of an investigation about the phenotypic assessment of growth and coloration dynamics as well as photosynthetic efficiency parameters in barley (*Hordeum vulgare*) HvASL (*Hordeum vulgare* albostrians-like) mutants and wildtype plants (Li et al., 2019). Barley HvASL mutants and wildtype plants were grown in a high throughput plant phenotyping facility for small plants. Seedlings of 9 different genotypes (7 mutants and 2 wild types) were grown for 15 days in single-plant setups in the automated phytochamber and imaged daily using RGB and static fluorescence imaging. Automated image analysis routines were employed for the extraction of growth-related features and coloration dynamics. Additionally, three times per week seedlings were subjected to kinetic chlorophyll fluorescence imaging and photosynthetic efficiency and quenching parameters were assessed in the light-adapted state as well as during induction of photosynthesis after transition from dark to light.

The dataset was encoded in ISA-Tab and is available as Junker & Li (2020).

Notes S1.4 Wheat dataset (GnpIS)

The wheat dataset is a subset of the Oury et al. (2018) dataset, focusing on 38 measures related to the quality of the grain for bread making on 10 wheat (*Triticum aestivum*) varieties. It includes 80 *studies* conducted from 2000-2014 over 8 experimental field locations in France to study the impact of nitrogen nutrition on several traits of interest in wheat production. In this dataset, each study represents one location over one year, as the *biological material* changes each year. All the *variables* were measured from a single sample of grains harvested from each variety and described using the Wheat INRA Phenotyping Ontology (WIPO) (Pommier et al.,

2019a). An *experimental factor* (named “itk”) is used to discriminate between nitrogen “treated”, “low nitrogen” input or “none”. All *biological material* is identified using accession numbers generated with the French Small Grain Cereals Genbank (Small Grain Genetic Resource Centre, 2020).

The wheat dataset is provided as an ISA-Tab archive at Oury et al. (2019a) and via the GnpIS BrAPI endpoint (Oury et al., 2019b).

Notes S1.5 Poplar dataset (GnpIS)

The poplar dataset is the result of the investigation detailed in Monclus et al. (2012) studying the variation of traits related to phenology, growth and water use efficiency in a full sib family of 360 poplar (*Populus trichocarpa* and *deltoides* crosses that produce the *Populus x generosa* species) individuals in three different locations. It corresponds to a test of clonal material (cuttings) derived from the same *material source* in orchards on three experimental sites in Europe over two years. The *material source* is the genbank accession and each *biological material* in each experimental site aggregates several individuals. The dataset has been organized in three two-year *studies*, which share the same *material source*. They include ten *observed variables* related to plant phenology, growth and water use efficiency, which are all included in the reference Woody Plant Ontology. *Ad hoc* variables were created in the dataset to refer to the measure of a given Woody Plant Ontology variable in different years as described in Pommier et al. (2019b). In this dataset, the trees were not identified by geographical coordinates, but by unique identifiers.

The poplar dataset is provided as an ISA-Tab archive at Michotey (2019), in the spreadsheet template in Supporting Information Archive S6, and via the GnpIS BrAPI endpoint (Michotey, 2020).

Notes S1.6 Maize datasets (VIB)

Three different maize (*Zea mays*) datasets were provided by VIB.

The first experiment details the assessment of variation in 103 lines of the maize B73xH99 recombinant inbred line (RIL) population in 13 studies in controlled growth chambers, for a set of primarily leaf size traits, complemented with measurements capturing growth dynamics, and cellular measurements (Baute et al., 2015). In the second experiment, 1,636 MAGIC maize RILs were derived from eight genetically diverse founder lines (Dell’Acqua et al., 2015). 529 of those lines were characterized, and a number of traits (ear height, plant height, pollen shedding and transformed grain yield) were determined in two fields. Finally, the third experiment describes the in-depth phenotyping of the fourth leaf at later stages of development in 197 RILs of two different maize populations (Baute et al., 2016). As a follow-up to the previous two experiments, the traits from the former were selected for assessment, which was conducted on the multiparent MAGIC population of the latter.

All three datasets are available via the VIB BrAPI endpoint (Baute et al., 2020, 2019c; Pea et al., 2019b), and in ISA-Tab format (produced via BrAPI2ISA) at Baute et al. (2019a), Pea et al. (2019a) and Baute et al. (2019b).

References

Arend D, Junker A, Scholz U, Schüler D, Wylie J, Lange M. **2016a**. PGP repository: a plant phenomics and genomics data publication infrastructure. *Database* **2016**: article ID baw033. doi: *10.1093/database/baw033*

Arend D, Lange M, Pape J-M, Weigelt-Fischer K, Arana-Ceballos F, Mücke I, Klukas C, Altmann T, Scholz U, Junker A. **2016b**. Quantitative monitoring of *Arabidopsis thaliana* growth and development using high-throughput plant phenotyping. *Scientific Data* **3**: article ID 160055. doi: *10.1038/sdata.2016.55*

Baute J, De Block J, Inzé D. **2019a**. *Zea mays* biparental RIL population - growth chamber phenotyping data [Data set]. *Zenodo.* doi: *10.5281/zenodo.3553692*

Baute J, De Block J, Inzé D. **2019b**. *Zea mays* MAGIC RIL population - growth chamber phenotyping data [Data set]. *Zenodo.* doi: *10.5281/zenodo.3553768*

Baute J, De Block J, Inzé D. **2020**. *Zea mays* biparental RIL population - growth chamber phenotyping data (PIPPA BrAPI endpoint) [Data set]. [WWW document] URL *https://pippa.psb.ugent.be/BrAPIPPA/brapi/v1/trials/1* [accessed 10 February 2020].

Baute J, De Block J, Inzé D. **2019c**. *Zea mays* MAGIC RIL population - growth chamber phenotyping data (PIPPA BrAPI endpoint) [Data set]. [WWW document] URL *https://pippa.psb.ugent.be/BrAPIPPA/brapi/v1/trials/2* [accessed 10 February 2020].

Baute J, Herman D, Coppens F, De Block J, Slabbinck B, Dell'Acqua M, Pè ME, Maere S, Nelissen H, Inzé D. **2015**. Correlation analysis of the transcriptome of growing leaves with mature leaf parameters in a maize RIL population. *Genome Biology* **16**: 168. doi: *10.1186/s13059-015-0735-9*

Baute J, Herman D, Coppens F, De Block J, Slabbinck B, Dell'Acqua M, Pè ME, Maere S, Nelissen H, Inzé D. **2016**. Combined Large-Scale Phenotyping and Transcriptomics in Maize Reveals a Robust Growth Regulatory Network. *Plant Physiology* **170** (**3**): 1848–1867. doi: *10.1104/pp.15.01883*

Chaves I, Miguel CM, Faria D, Costa BV. **2019a**. Enabling reusability of plant phenomic datasets with MIAPPE 1.1 - Supplementary dataset iBET [Data set]. *Portail Data INRAE.* doi: 10.15454/AH6U4A

Chaves I, Miguel CM, Faria D, Costa BV. **2019b**. Enabling reusability of plant phenomic datasets with MIAPPE 1.1 - Supplementary dataset iBET (PHENO BrAPI endpoint) [Data set]. [WWW document] URL *https://brapi.biodata.pt/brapi/v1/trials/2* [accessed 10 February 2020].

Costa A, Barbosa I, Roussado C, Graça J, Spiecker H. **2016**. Climate response of cork growth in the Mediterranean oak (*Quercus suber L.*) woodlands of southwestern Portugal. *Dendrochronologia* **38**: 72–81. doi: *10.1016/j.dendro.2016.03.007*

Dell'Acqua M, Gatti DM, Pea G, Cattonaro F, Coppens F, Magris G, Hlaing AL, Aung HH, Nelissen H, Baute J et al. 2015. Genetic properties of the MAGIC maize population: a new platform for high definition QTL mapping in *Zea mays*. *Genome Biology* **16**: 167. doi: *10.1186/s13059-015-0716-z*

Inácio V, Barros PM, Costa A, Roussado C, Gonçalves E, Costa R, Graça J, Oliveira MM, Morais-Cecílio L. **2017**. Differential DNA methylation patterns are related to phellogen origin and quality of *Quercus suber* cork. *PLoS One* **12** (**1**): e0169018. doi: *10.1371/journal.pone.0169018*

Junker A. **2020**. Raw images files from quantitative monitoring of 484 *Arabidopsis thaliana* plants using high-throughput plant phenotyping (MIAPPE 1.1 update) [Data set]. *e!DAL - Plant Genomics and Phenomics Research Data Repository (PGP), IPK Gatersleben, Seeland OT Gatersleben, Corrensstraße 3, 06466, Germany.* doi: *10.5447/IPK/2020/3*

Junker A, Li M. **2020**. Phenotypic assessment of growth and coloration dynamics as well as photosynthetic efficiency parameters in barley HvASL mutants and wild type plants [Data set]. *e!DAL - Plant Genomics and Phenomics Research Data Repository (PGP), IPK Gatersleben, Seeland OT Gatersleben, Corrensstraße 3, 06466, Germany.* doi: *10.5447/IPK/2020/4*

Junker A, Muraya MM, Weigelt-Fischer K, Arana-Ceballos F, Klukas C, Melchinger AE, Meyer RC, Riewe D, Altmann T. **2015**. Optimizing experimental procedures for quantitative evaluation of crop plant performance in high throughput phenotyping systems. *Frontiers in Plant Science* **5**: 770. doi: *10.3389/fpls.2014.00770*

Junker A, Weigelt-Fischer K, Altmann T, Klukas C. **2020**. Raw images files from quantitative monitoring of 484 *Arabidopsis thaliana* plants using high-throughput plant phenotyping [Data set]. *e!DAL - Plant Genomics and Phenomics Research Data Repository (PGP), IPK Gatersleben, Seeland OT Gatersleben, Corrensstraße 3, 06466, Germany.* doi: *10.5447/IPK/2016/7*

Li M, Hensel G, Melzer M, Junker A, Tschiersch H, Arend D, Kumlehn J, Börner T, Stein N. **2019**. Mutation of the ALBOSTRIANS Ohnologous Gene HvCMF3 Impairs Chloroplast Development and Thylakoid Architecture in Barley due to Reduced Plastid Translation. *bioRxiv*: 756833. doi: *10.1101/756833*

Michotey C. **2019**. Integrating genome annotation and QTL position to identify candidate genes for productivity, architecture and water-use efficiency in *Populus* spp - Supplementary dataset [Data set]. *Portail Data INRAE.* doi: *10.15454/EASUQV*

Michotey C. **2020**. Integrating genome annotation and QTL position to identify candidate genes for productivity, architecture and water-use efficiency in *Populus* spp - Supplementary dataset (GnpIS BrAPI endpoint) [Data set]. [WWW document] URL *https://urgi.versailles.inra.fr/faidare/brapi/v1/trials/aHR0cDovL2R4LmRvaS5vcmcvMTAuMTE4Ni8xNDcxLTIyMjktMTItMTcz* [accessed 10 February 2020].

Michotey C, Chaves I. **2020**. Woody Plant Ontology. [WWW document] URL *http://www.cropontology.org/ontology/CO_357/Woody%20Plant%20Ontology* [accessed 10 February 2020].

Michotey C, Chaves I, Anger C, Jorge V, Ehrenmann F, Jean F, Opgenoorth L. **2019**. Woody Plant Ontology. *Portail Data INRAE, V1.* doi: *10.15454/JB2WCE*

Monclus R, Leplé J-C, Bastien C, Bert P-F, Villar M, Marron N, Brignolas F, Jorge V. **2012**. Integrating genome annotation and QTL position to identify candidate genes for productivity, architecture and water-use efficiency in *Populus spp*. *BMC Plant Biology* **12**: 173. doi: *10.1186/1471-2229-12-173*

Oury F-X, Heumez E, Rolland B, Auzanneau J, Bérard P, Brancourt-Hulmel M, Charrier X, Chiron H, Depatureaux C, Falchetto L et al. 2018. Winter wheat (*Triticum aestivum L*) phenotypic data from the multiannual, multilocal field trials of the INRA Small Grain Cereals Network. *Portail Data INRAE, V5.* doi: *10.15454/1.4489666216568333E12*

Oury F-X, Pommier C, Charmet G. **2019a**. Enabling reusability of plant phenomic datasets with MIAPPE 1.1 - Supplementary dataset INRA Wheat [Data set]. *Portail Data INRAE.* doi: *10.15454/1AFKZ2*

Oury F-X, Pommier C, Charmet G. **2019b**. Enabling reusability of plant phenomic datasets with MIAPPE 1.1 - Supplementary dataset INRA Wheat (GnpIS BrAPI endpoint) [Data set]. [WWW document] URL *https://urgi.versailles.inra.fr/faidare/brapi/v1/trials/dXJuOlVSR0kvdHJpYWwvNw==* [accessed 10 February 2020].

Papoutsoglou EA, Faria D, Arend D, Arnaud E, Athanasiadis IN, Chaves I, Coppens F, Cornut G, Costa BV, Ćwiek-Kupczyńska H et al. 2019. Enabling reusability of plant phenomic datasets with MIAPPE 1.1 - Supplementary dataset. *Portail Data INRAE.* doi: *10.15454/1YXVZV*

Pea G, Hlaing AL, Aung HH, Pè ME. **2019a**. *Zea mays* MAGIC RIL population - field trial phenotyping data [Data set]. *Zenodo.* doi: *10.5281/zenodo.3553749*

Pea G, Hlaing AL, Aung HH, Pè ME. **2019b**. *Zea mays* MAGIC RIL population - field trial phenotyping data (PIPPA BrAPI endpoint) [Data set]. [WWW document] URL *https://pippa.psb.ugent.be/BrAPIPPA/brapi/v1/trials/3* [accessed 10 February 2020].

Pommier C, Letellier T, Pietragalla J, Laporte MA, Arnaud E, Le Gouis J, Shrestha R. **2019a**. Wheat Crop Ontology. *Portail Data INRAE, V1.* doi: *10.15454/3EDMCP*

Pommier C, Michotey C, Cornut G, Roumet P, Duchêne E, Flores R, Lebreton A, Alaux M, Durand S, Kimmel E et al. 2019b. Applying FAIR Principles to Plant Phenotypic Data Management in GnpIS. *Plant Phenomics* **2019**: article ID 1671403. doi: *10.34133/2019/1671403*

Small Grain Genetic Resource Centre. **2020**. Small Grain Genetic Resource Centre. [WWW document] URL *https://urgi.versailles.inra.fr/siregal/siregal/card.do?id=1&dbName=siregal&className=genres.administration.GrcImpl* [accessed 10 February 2020].
