# Supplementary material for: Enabling reusability of plant phenomic datasets with MIAPPE 1.1
Source: New Phytol. 2020 Apr 25;227(1):260–73. doi: 10.1111/nph.16544 (PMC7317793; doi:10.1111/nph.16544)
Supplement: Supplementary file 2 — Table S1 Detailed mapping between MIAPPE, ISA‐Tab and BrAPI fields. Please note: Wiley Blackwell are not responsible for the content or functionality of any Supporting Information supplied by the authors. Any queries (other than missing material) should be directed to the New Phytologist Central Office. [file NPH-227-260-s002.pdf]

New Phytologist Supporting Information

Article title: Enabling reusability of plant phenomic datasets with MIAPPE 1.1

Authors:
Evangelia A. Papoutsoglou, Daniel Faria, Daniel Arend, Elizabeth Arnaud, Ioannis N. Athanasiadis, Inês Chaves, Frederik Coppens, Guillaume Cornut, Bruno V. Costa,
Hanna Ćwiek-Kupczyńska, Bert Droesbeke, Richard Finkers, Kristina Gruden, Astrid Junker, Graham J. King, Paweł Krajewski, Matthias Lange, Marie-Angélique Laporte,
Célia Michotey, Markus Oppermann, Richard Ostler, Hendrik Poorter, Ricardo Ramirez-Gonzalez, Živa Ramšak Jochen C. Reif, Philippe Rocca-Serra, Susanna-Assunta Sansone,
Uwe Scholz, François Tardieu, Cristobal Uauy, Björn Usadel, Richard G.F. Visser, Stephan Weise, Paul J. Kersey, Célia M. Miguel, Anne-Françoise Adam-Blondon, Cyril Pommier

Article acceptance date: 24 February 2020.

Table S1. Detailed mapping between MIAPPE, ISA-Tab and BrAPI fields.

	MIAPPE	ISA-Tab			BrAPI		
line #	MIAPPE Check list	ISA-Tab File	ISA-Tab Section (for Investigation file)	ISA-Tab Field	BrAPI Call	BrAPI Object(s)	BrAPI Field(s)
DM-1	**Investigation**						
DM-2	Investigation unique ID	Investigation	INVESTIGATION	Investigation Identifier	/trials/{trialDbId}	None	trialDbId
DM-3	Investigation title	Investigation	INVESTIGATION	Investigation Title	/trials/{trialDbId}	None	trialName
DM-4	Investigation description	Investigation	INVESTIGATION	Investigation Description	/trials/{trialDbId}	None	trialDescription
DM-5	Submission date	Investigation	INVESTIGATION	Investigation Submission Date	/trials/{trialDbId}	datasetAuthorships	submissionDate
DM-6	Public release date	Investigation	INVESTIGATION	Investigation Public Release Date	/trials/{trialDbId}	datasetAuthorships	publicReleaseDate
DM-7	License	Investigation	INVESTIGATION	Comment[License]	/trials/{trialDbId}	datasetAuthorships	license
DM-8	MIAPPE version	Investigation	INVESTIGATION	Comment[MIAPPE version]	*out of scope*		
DM-9	Associated publication	Investigation	INVESTIGATION PUBLICATIONS	Investigation Publication DOI	/trials/{trialDbId}	publications	publicationPUI
DM-10	**Study**						
DM-11	Study unique ID	Investigation	STUDY	Study Identifier	/studies/{studyDbId}	None	studyDbId
DM-12	Study title	Investigation	STUDY	Study Title	/studies/{studyDbId}	None	studyName
DM-13	Study description	Investigation	STUDY	Study Description	/studies/{studyDbId}	None	studyDescription
DM-14	Start date of study	Investigation	STUDY	Comment[Study Start Date]	/studies/{studyDbId}	None	startDate
DM-15	End date of study	Investigation	STUDY	Comment[Study End Date]	/studies/{studyDbId}	None	endDate
DM-16	Contact institution	Investigation	STUDY	Comment[Study Contact Institution]	/studies/{studyDbId}	None	instituteName
DM-17	Geographic location (country)	Investigation	STUDY	Comment[Study Country]	/studies/{studyDbId}	location	countryName / countryCode
DM-18	Experimental site name	Investigation	STUDY	Comment[Study Experimental Site]	/studies/{studyDbId}	location	name
DM-19	Geographic location (latitude)	Investigation	STUDY	Comment[Study Latitude]	/studies/{studyDbId}	location	latitude
DM-20	Geographic location (longitude)	Investigation	STUDY	Comment[Study Longitude]	/studies/{studyDbId}	location	longitude
DM-21	Geographic location (altitude)	Investigation	STUDY	Comment[Study Altitude]	/studies/{studyDbId}	location	altitude
DM-22	Description of the experimental design	Investigation	STUDY DESIGN DESCRIPTORS	Comment[Study Design Description]	/studies/{studyDbId}	experimentalDesign	description
DM-23	Type of experimental design	Investigation	STUDY DESIGN DESCRIPTORS	Study Design Type	/studies/{studyDbId}	experimentalDesign	PUI
DM-24	Observation unit level hierarchy	Investigation	STUDY DESIGN DESCRIPTORS	Comment[Observation Unit Level Hierarchy]	/studies/{studyDbId}	additionalInfo	observationUnitLevelHierarchy
DM-25	Observation unit description	Investigation	STUDY DESIGN	Comment[Observation Unit Description]	/studies/{studyDbId}	None	observationUnitsDescription
DM-26	Description of growth facility	Investigation	STUDY DESIGN DESCRIPTORS	Comment[Description of Growth Facility]	/studies/{studyDbId}	growthFacility	description
DM-27	Type of growth facility	Investigation	STUDY DESIGN DESCRIPTORS	Comment[Type of Growth Facility]	/studies/{studyDbId}	growthFacility	PUI
DM-28	Cultural practices	Investigation	STUDY PROTOCOLS	Study Protocol Description (for Growth protocol)	/studies/{studyDbId}	None	culturalPractices
DM-29	Map of experimental design	Investigation	STUDY DESIGN DESCRIPTORS	Comment[Map of Experimental Design]	/studies/{studyDbId}	additionalInfo	mapOfExperimentalDesign
DM-30	**Person**						
DM-31	Person name	Investigation	INVESTIGATION CONTACTS / STUDY CONTACTS	Investigation Person Last Name - First Name - Mid Initials / Study Person Last Name - First Name - Mid Initials	/studies/{studyDbId}	contacts	name

DM-32	Person email	Investigation	INVESTIGATION CONTACTS / STUDY CONTACTS	Investigation Person Email / Study Person Email	/studies/{studyDbId}	contacts	email
DM-33	Person ID	Investigation	INVESTIGATION CONTACTS / STUDY CONTACTS	Comment[Person ID]	/studies/{studyDbId}	contacts	orcid / contactDbId
DM-34	Person role	Investigation	INVESTIGATION CONTACTS / STUDY CONTACTS	Investigation Person Roles / Study Person Roles	/studies/{studyDbId}	contacts	type
DM-35	Person affiliation	Investigation	INVESTIGATION CONTACTS / STUDY CONTACTS	Investigation Person Affiliation / Study Person Affiliation	/studies/{studyDbId}	contacts	instituteName
DM-36	**Data File**						
DM-37	Data file link	Investigation	STUDY	Comment[Study Data File Link]	/studies/{studyDbId}	dataLinks	type
DM-38	Data file description	Investigation	STUDY	Comment[Study Data File Description]	/studies/{studyDbId}	dataLinks	name / url
DM-39	Data file version	Investigation	STUDY	Comment[Study Data File Version]	/studies/{studyDbId}	dataLinks	version
DM-40	**Biological Material**						
DM-41	Biological material ID	Study	None	Source Name	/germplasm/{germplasmDbId}	None	accessionNumber
DM-42	Organism	Study	Source	Characteristics[Organism]	/germplasm/{germplasmDbId}	taxonIds	sourceName, taxonId
DM-43	Genus	Study	Source	Characteristics[Genus]	/germplasm/{germplasmDbId}	None	germplasmGenus
DM-44	Species	Study	Source	Characteristics[Species]	/germplasm/{germplasmDbId}	None	germplasmSpecies
DM-44	Infraspecific name	Study	Source	Characteristics[Infraspecific Name]	/germplasm/{germplasmDbId}	None	subtaxa
DM-45	Biological material latitude	Study	Source	Characteristics[Biological Material Latitude]	/germplasm/{germplasmDbId}	germplasmOrigin	latitudeDecimal
DM-46	Biological material longitude	Study	Source	Characteristics[Biological Material Longitude]	/germplasm/{germplasmDbId}	germplasmOrigin	longitudeDecimal
DM-47	Biological material altitude	Study	Source	Characteristics[Biological Material Altitude]	/germplasm/{germplasmDbId}	germplasmOrigin	altitude
DM-48	Biological material coordinates uncertainty	Study	Source	Characteristics[Biological Material Coordinates Uncertainty]	/germplasm/{germplasmDbId}	germplasmOrigin	coordinateUncertainty
DM-49	Biological material preprocessing	Study	Source	Characteristics[Biological Material Preprocessing]	/germplasm/{germplasmDbId}	None	germplasmPreprocessing
DM-50	Material source ID (Holding institute/stock centre, accession)	Study	Source	Characteristics[Material Source ID]	/germplasm/{germplasmDbId}/mcpd	donorInfo	donorAccessionNumber
DM-51	Material source DOI	Study	Source	Characteristics[Material Source DOI]	/germplasm/{germplasmDbId}/mcpd	donorInfo	donorAccessionPui
DM-52	Material source latitude	Study	Source	Characteristics[Material Source Latitude]	/germplasm/{germplasmDbId}/mcpd	collectingInfo.collectingSite	latitudeDecimal
DM-53	Material source longitude	Study	Source	Characteristics[Material Source Longitude]	/germplasm/{germplasmDbId}/mcpd	collectingInfo.collectingSite	longitudeDecimal
DM-54	Material source altitude	Study	Source	Characteristics[Material Source Altitude]	/germplasm/{germplasmDbId}/mcpd	collectingInfo.collectingSite	elevation
DM-55	Material source coordinates uncertainty	Study	Source	Characteristics[Material Source Coordinates Uncertainty]	/germplasm/{germplasmDbId}/mcpd	collectingInfo.collectingSite	coordinateUncertainty
DM-56	Material source description	Study	Source	Characteristics[Material Source Description]	/germplasm/{germplasmDbId}	None	seedSourceDescription
DM-57	**Environment**						
DM-58	Environment parameter	Investigation	STUDY PROTOCOLS	Study Protocol Parameters Name *(for Growth protocol)*	/studies/{studyDbId}	environmentParameters	parameterName
DM-59	Environment parameter value	Study	Growth protocol	Parameter Value[ ]	/studies/{studyDbId}	environmentParameters	description
DM-60	**Experimental Factor**						
DM-61	Experimental Factor type	Investigation	STUDY FACTORS	Study Factor Name	/observationunits	Treatment	Factor
DM-62	Experimental Factor description	Investigation	STUDY FACTORS	Comment[Study Factor Description]	None	None	None
DM-63	Experimental Factor values	Investigation	STUDY FACTORS	Comment[Study Factor Values]	out of scope		
DM-64	**Event**						
DM-65	Event type	Investigation	STUDY PROTOCOLS	Study Protocol Name *(for protocol of type Event)*	/events	None	eventTypeName
DM-66	Event accession number	Investigation	STUDY PROTOCOLS	Study Protocol URI *(for protocol of type Event)*	/events	None	eventTypeDbId
DM-67	Event description	Investigation	STUDY PROTOCOLS	Study Protocol Description *(for protocol of type Event)*	/events	None	description
DM-68	Event date	Event file	None	Event Date	/events	None	date
DM-69	**Observation Unit**						
DM-70	Observation unit ID	Study / Assay	None	Sample Name	/observationunits	None	observationUnitDbId
DM-71	Observation unit type	Study	Sample	Characteristics[Observation Unit Type]	/observationunits	None	observationLevel

DM-72	External ID	Study	Sample	Characteristics[External ID]	/observationunits	observationUnitXref	id/source
DM-73	Spatial distribution	Study	Sample	Characteristics[Spatial distribution]	/observationunits	None	observationLevels
DM-74	Observation Unit factor value	Study	Source / Sample	Factor Value[ ]	/observationunits	treatments	factor/modality
DM-75	**Sample**						
DM-76	Sample ID	Assay	None	Extract Name	/samples	None	sampleDbId
DM-77	Plant structure development stage	Assay	Extract	Characteristics[Plant Structure Development Stage]	/samples	additionalInfo	plantStructureDevelopmentStage
DM-78	Plant anatomical entity	Assay	Extract	Characteristics[Plant Anatomical Entity]	/samples	None	tissueType
DM-79	Sample description	Assay	Sampling protocol	Parameter Value[Sampling Description]	/samples	additionalInfo	samplingDescription
DM-80	Collection date	Assay	Sampling protocol	Parameter Value[Sampling Date]	/samples	None	sampleTimestamp
DM-81	External ID	Assay	Extract	Characteristics[External ID]	/samples	additionalInfo	externalId
DM-82	**Observed Variable**						
DM-83	Variable ID	Trait Definition File	None	Variable ID	/variables	None	observationVariableName
DM-84	Variable name	Trait Definition File	None	Variable name	/variables	None	observationVariableName, observationVariableDbId
DM-85	Variable accession number	Trait Definition File	None	Variable accession number	/variables	None	xref, (observationVariableDbId)
DM-86	Trait	Trait Definition File	None	Trait	/variables	trait	traitName, description
DM-87	Trait accession number	Trait Definition File	None	Trait accession number	/variables	trait	(traitDbId)
DM-88	Method	Trait Definition File	None	Method	/variables	method	methodName
DM-89	Method accession number	Trait Definition File	None	Method accession number	/variables	method	(methodDbId)
DM-90	Method description	Trait Definition File	None	Method description	/variables	method	description
DM-91	Reference associated to the method	Trait Definition File	None	Reference associated to the method	/variables	method	reference
DM-92	Scale	Trait Definition File	None	Scale	/variables	scale	scaleName
DM-93	Scale accession number	Trait Definition File	None	Scale accession number	/variables	scale	(scaleDbId)
DM-94	Time scale	Trait Definition File	None	Time scale	None	None	None
